# Flush Occlusion of the Superficial Femoral Artery Managed Using a Retrograde Popliteal Approach: A Case Report

**DOI:** 10.7759/cureus.95224

**Published:** 2025-10-23

**Authors:** Nadeen Aladham, Herbert Oye

**Affiliations:** 1 Vascular Surgery, Ross University School of Medicine, Miramar, USA; 2 Surgery/Endovascular Surgery, Raleigh General Hospital, Beckley, USA; 3 Vascular Surgery, West Virginia Vascular Institute, Beckley, USA

**Keywords:** drug-coated balloon, endovascular revascularization, femoral popliteal bypass, flush occlusion, peripheral arterial disease, retrograde popliteal approach, superficial femoral artery

## Abstract

Flush occlusion of the superficial femoral artery (SFA) poses significant challenges for endovascular revascularization because the absence of a distal stump complicates antegrade access. Traditional treatment options include balloon-directed catheter thrombectomy, mechanical thrombectomy, and tissue plasminogen activator thrombolysis. However, these approaches may be less effective in cases of flush or long-segment occlusions. The retrograde popliteal approach has emerged as a viable alternative, providing direct access to the distal true lumen and proving particularly useful when antegrade strategies fail. This case report describes the successful use of the retrograde popliteal approach to treat a flush SFA occlusion caused by arterial thrombosis, highlighting its role in managing complex peripheral arterial disease.

## Introduction

Lower extremity ischemia caused by superficial femoral artery (SFA) occlusion can result in significant morbidity if not promptly managed. Arterial thrombosis, often superimposed on atherosclerotic plaque, is a common underlying mechanism. Thrombus at the origin of the profunda femoris artery frequently arises from a combination of proximal plaque rupture or ulceration, distal embolization, and local flow stagnation. In flush SFA occlusion, altered hemodynamics at the femoral bifurcation can predispose to thrombus formation within the profunda origin.

Treatment options include Fogarty catheter thrombectomy, mechanical thrombectomy, tissue plasminogen activator thrombolysis, and conventional antegrade endovascular revascularization [1-3]. However, certain lesions, particularly flush or long-segment occlusions, pose technical challenges that may limit the success of antegrade approaches.

The retrograde popliteal approach provides direct access to the distal true lumen and has emerged as a reliable alternative in these difficult cases. Thrombus and atheromatous debris were removed using manual aspiration through selective catheters and mechanical extraction with a Fogarty thrombectomy catheter when required. When endovascular aspiration alone was insufficient, an open popliteal arteriotomy allowed direct Fogarty thrombectomy and facilitated retrograde wiring. Balloon angioplasty was then used for vessel preparation, followed by a drug-coated balloon to treat the underlying atherosclerotic disease.

Modern techniques employing ultrasound guidance and low-profile devices have improved both safety and technical success. Studies report high technical success rates and favorable short- to mid-term patency outcomes [4-6]. This approach is particularly valuable when traditional antegrade, thrombectomy, or thrombolysis strategies are unsuccessful, offering a viable solution for complex SFA occlusions. In this patient, a less invasive strategy was favored due to comorbidities and the technical feasibility of retrograde popliteal access.

## Case presentation

A 56-year-old female with a history of hypertension, hyperlipidemia, hypothyroidism, and peripheral vascular disease presented with acute left leg pain. She denied chest pain, dizziness, or syncope. On physical examination, distal pulses in the left lower extremity were diminished compared with the contralateral side.

Laboratory findings on presentation are summarized in Table 1, with reference ranges derived from Mayo Clinic Laboratories and institutional norms [7].

**Table 1:** Laboratory findings on presentation

Test	Patient value	Reference range	Unit
White blood cell count	7.9	4.0-11.0	× 10⁹/L
Hemoglobin	13.8	11.6-15.0	g/dL
Hematocrit	42.5	35-45	%
Platelet count	237	150-450	× 10⁹/L
Sodium	140	135-145	mmol/L
Potassium	3.8	3.5-5.0	mmol/L
Chloride	108	98-107	mmol/L
Bicarbonate	23	22-29	mmol/L
Blood urea nitrogen	15	7-20	mg/dL
Creatinine	0.8	0.59-1.04	mg/dL
Glucose	97	70-100	mg/dL

**Figure 1:**
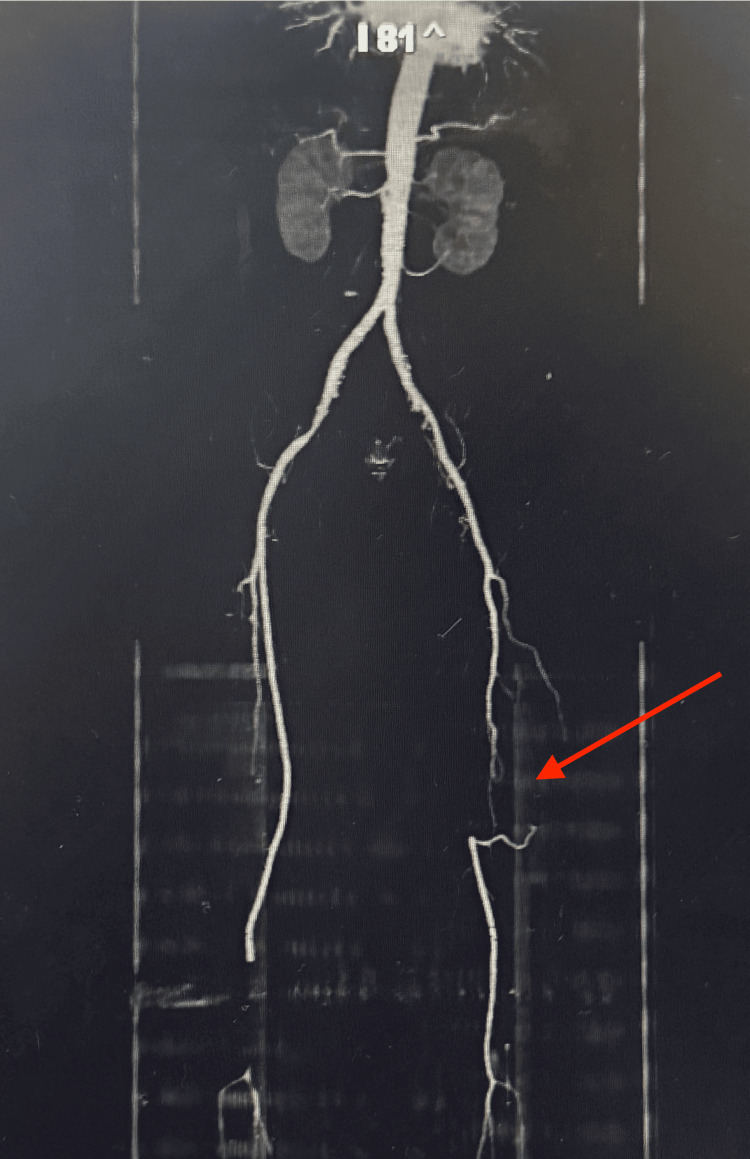
CT angiogram with runoff demonstrating complete occlusion of the left SFA (red arrow) SFA, superficial femoral artery

Abdominal CT angiography (CTA) with runoff demonstrated complete occlusion of the left SFA (Figure 1). No other significant vascular abnormalities were identified.

**Figure 2:**
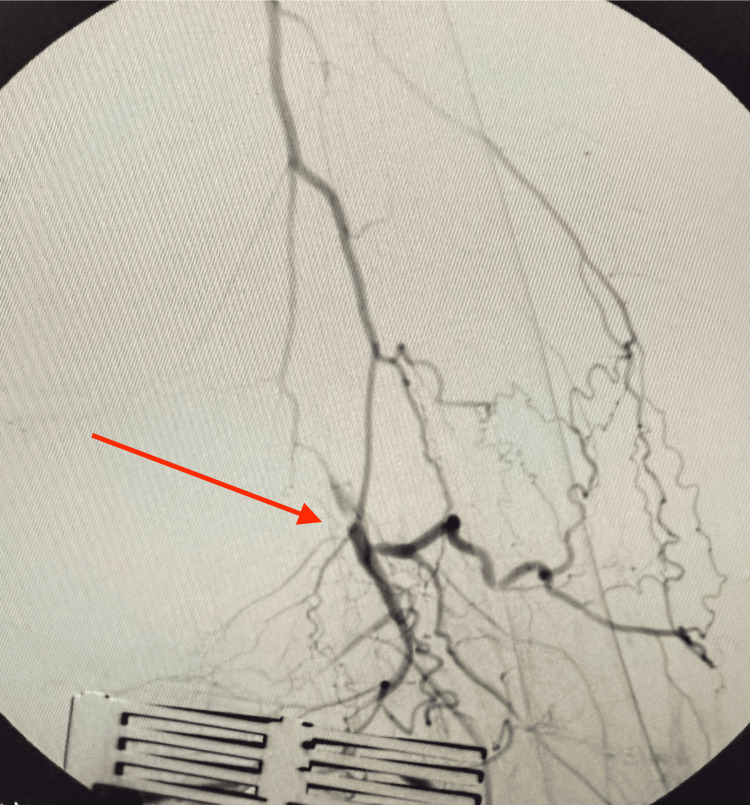
Fluoroscopic angiogram of the left SFA showing complete occlusion (red arrow) SFA, superficial femoral artery

Given the CTA findings, informed consent was obtained, and the patient was prepared for surgery. An angiogram with fluoroscopy confirmed complete occlusion of the left SFA (Figure 2).

**Figure 3:**
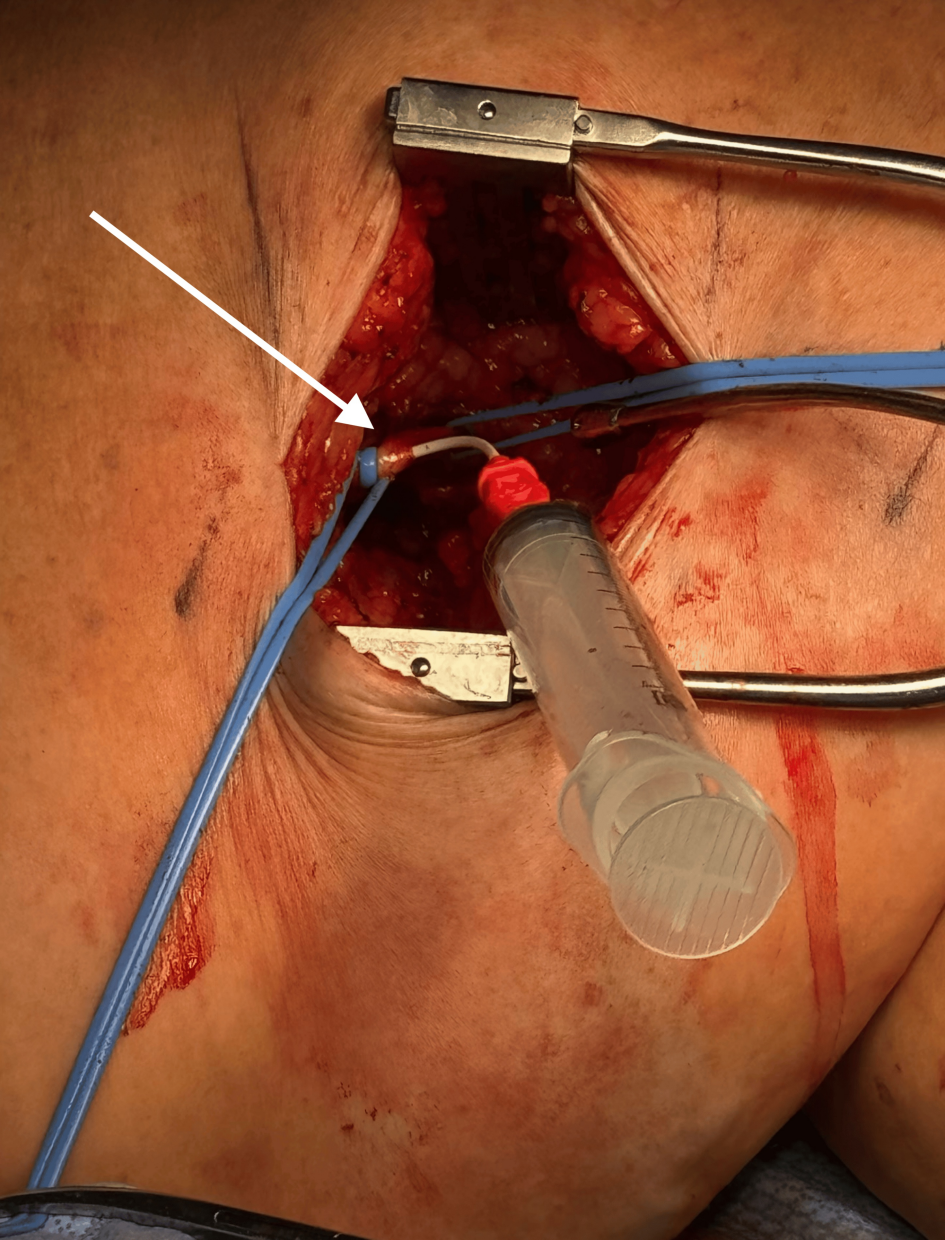
Exposure of the left popliteal artery for retrograde access (white arrow)

An initial attempt to cross the lesion via a right femoral approach with a crossover sheath was unsuccessful. Given the failure of antegrade access, the decision was made to expose the left popliteal artery to allow retrograde cannulation of the popliteal, SFA, and iliac arteries (Figure 3).

**Figure 4:**
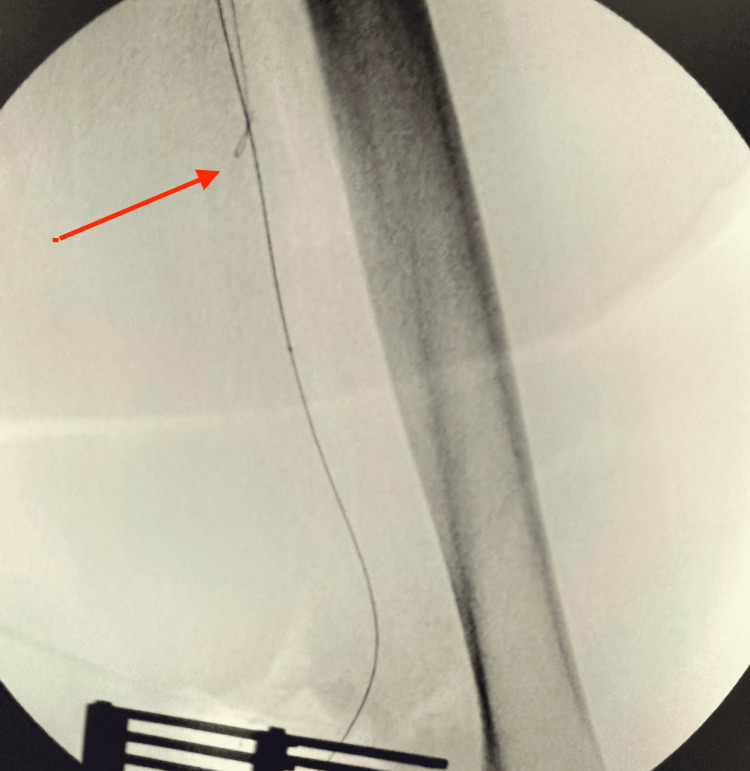
Site of complete arterial occlusion, with the guidewire attempting to cross the blockage (red arrow)

**Figure 5:**
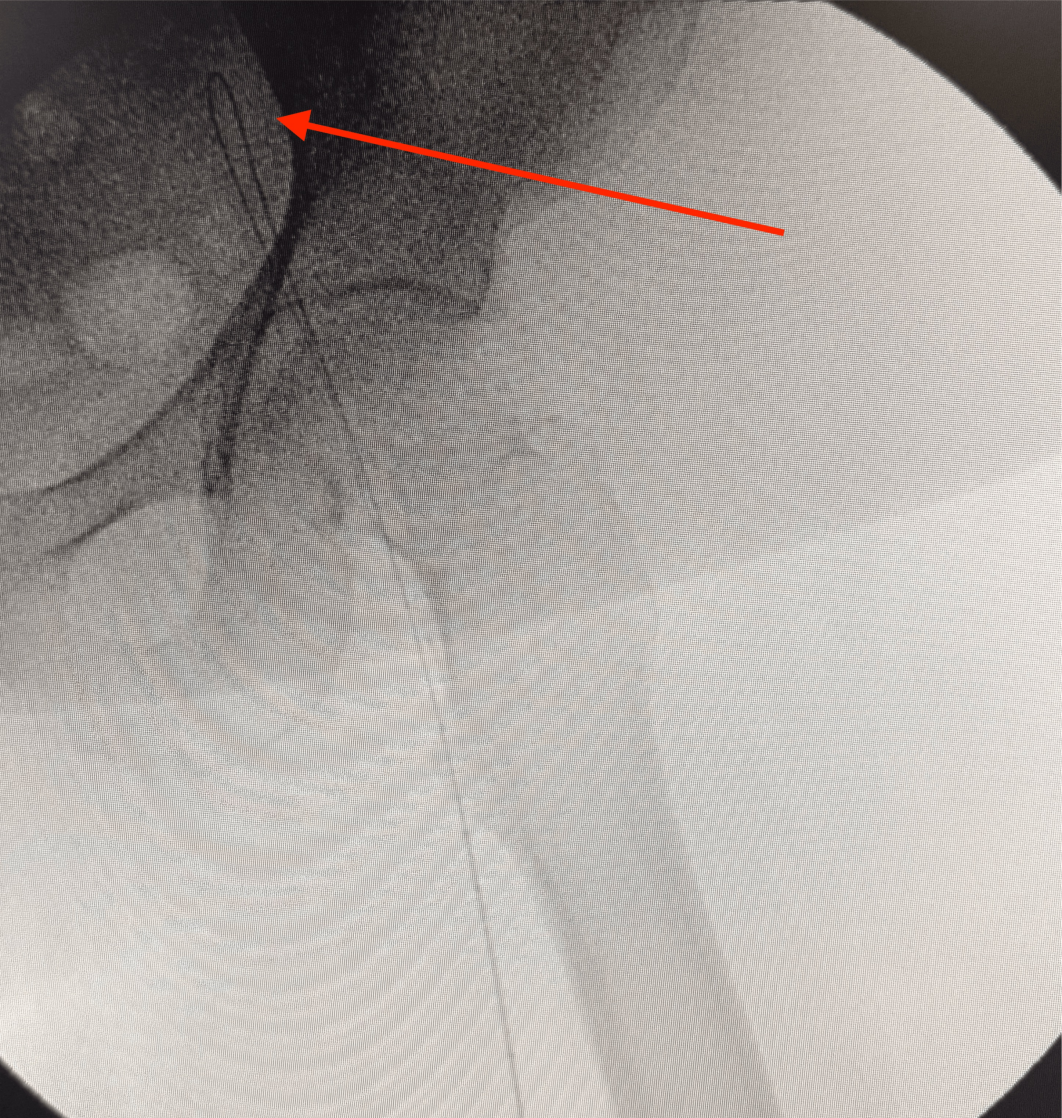
Continued advancement of the wire, attempting to traverse the occluded segment (red arrow)

**Figure 6:**
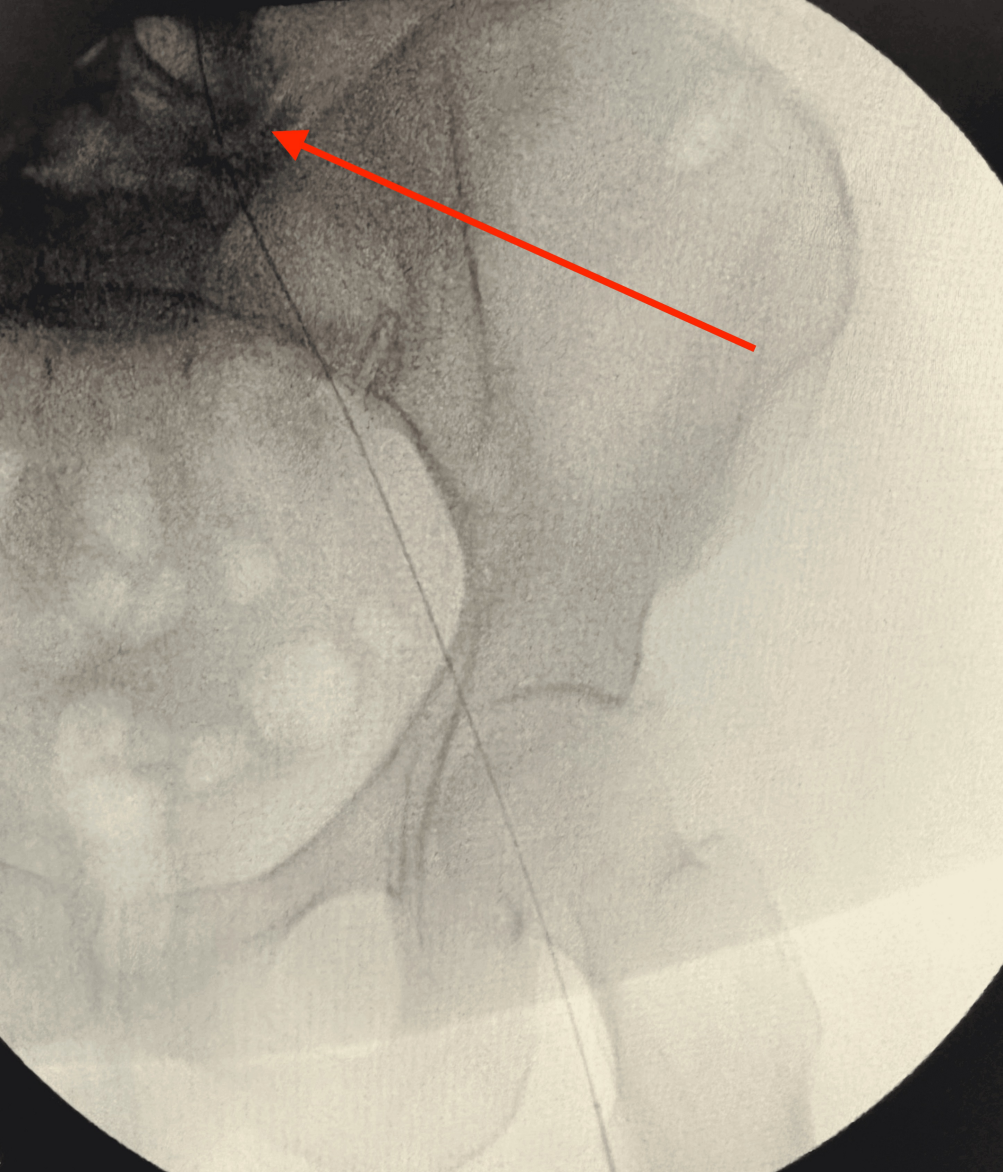
Wire successfully advanced to the iliac artery (red arrow)

**Figure 7:**
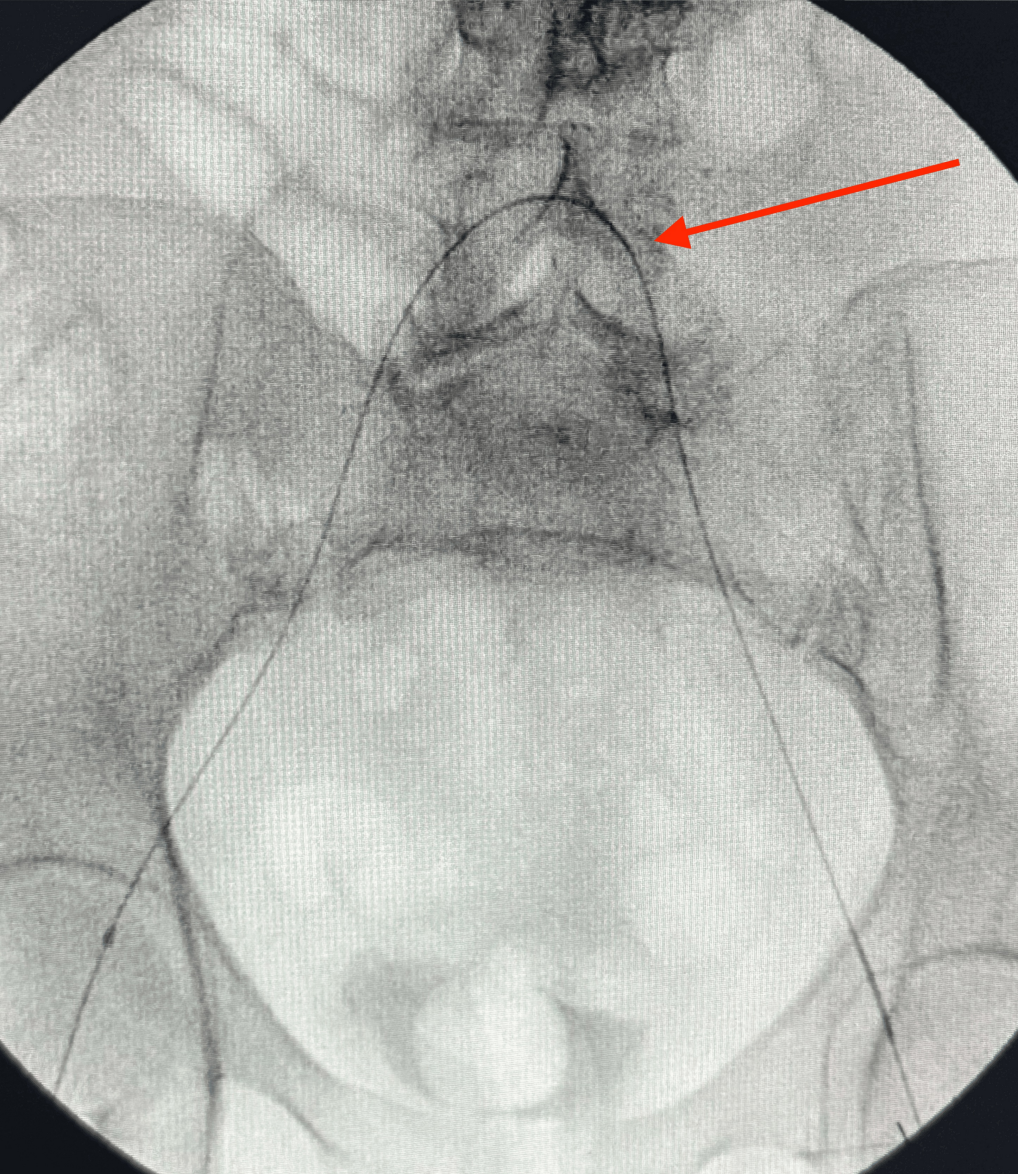
Recanalization of the iliac artery (red arrow)

Through a popliteal arteriotomy, the lesion was successfully crossed retrogradely. A stepwise retrograde popliteal approach was performed for revascularization of the completely occluded left SFA. The site of complete arterial occlusion is shown with the guidewire attempting to cross the blockage (Figure 4). Continued advancement of the wire is illustrated as it traverses the occluded segment (Figure 5), eventually reaching the iliac artery (Figure 6). Subsequent fluoroscopic imaging confirmed a patent left iliac artery (Figure 7).

**Figure 8:**
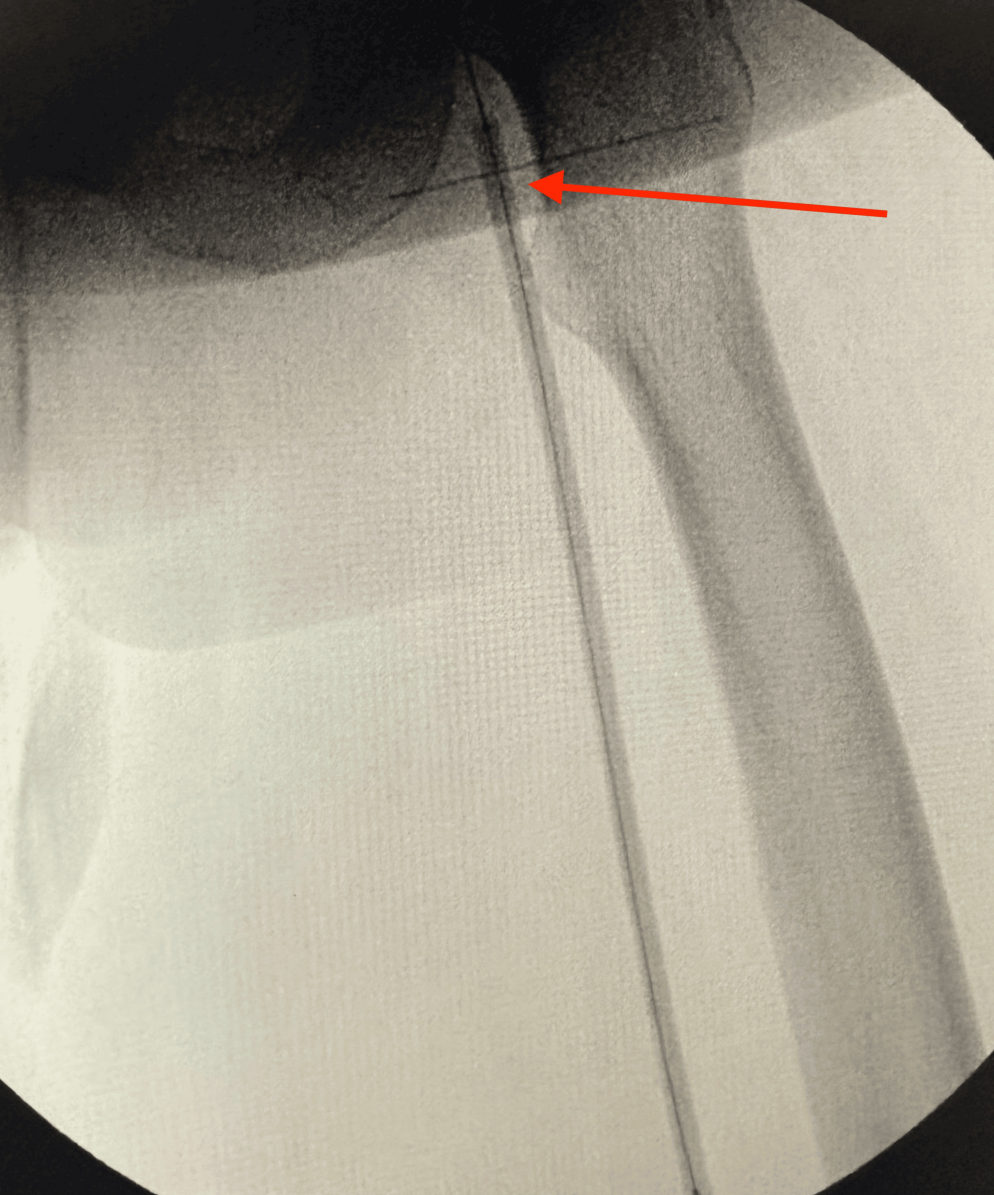
First balloon positioned at the level where the flush occlusion begins (red arrow)

**Figure 9:**
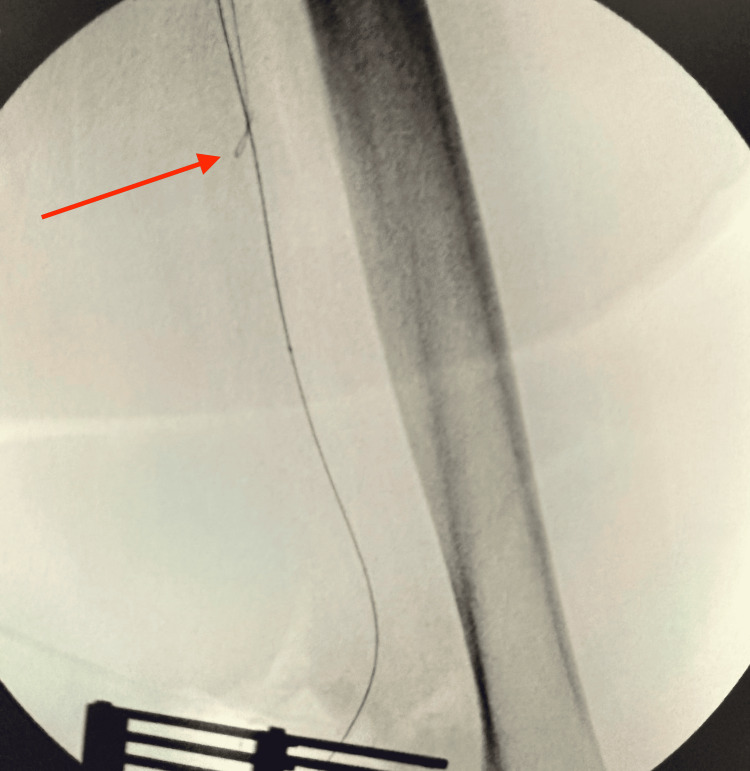
Guidewire advancing toward the occlusion, approaching the level of the blockage (red arrow)

**Figure 10:**
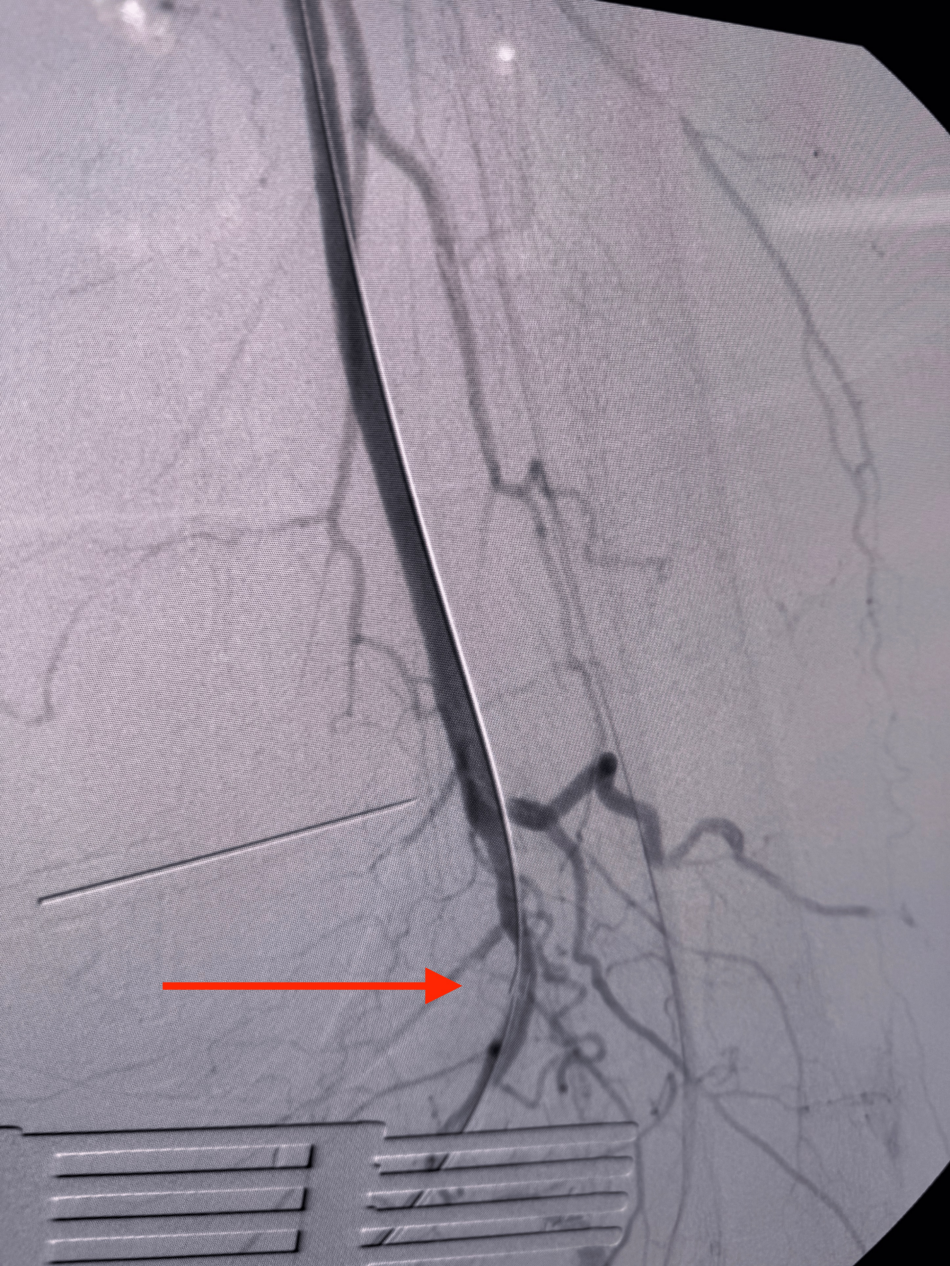
Balloon advanced from the groin toward the occlusion site, with contrast outlining its position (red arrow)

**Figure 11:**
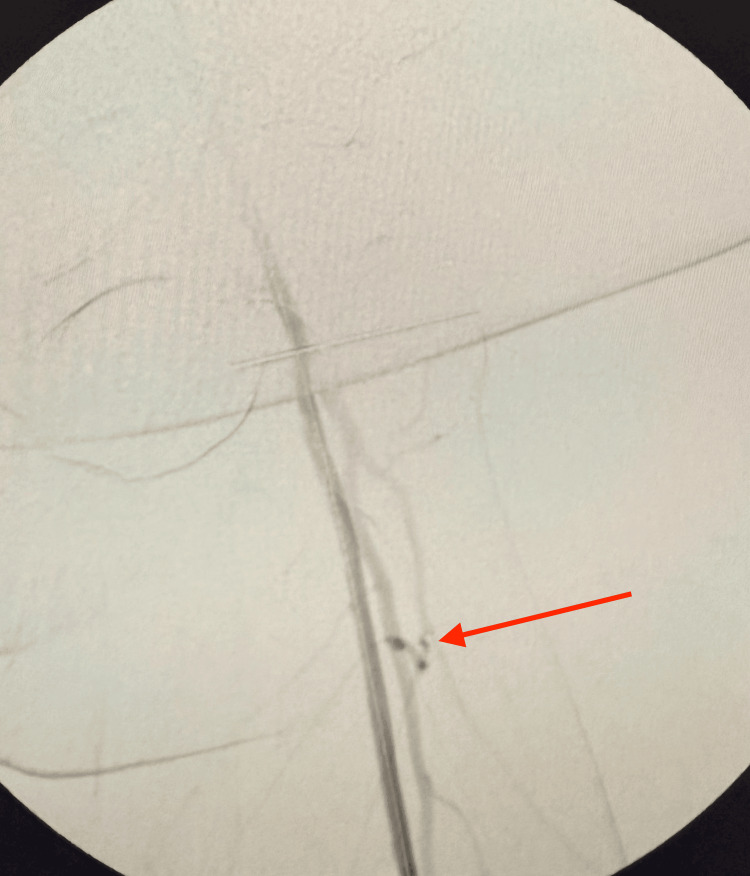
Flush occlusion successfully opened, with thrombus noted at the origin of the profunda femoris artery (red arrow)

The first balloon was positioned at the level where the flush occlusion begins (Figure 8). The guidewire continued to advance toward the occlusion and approached the level of the blockage (Figure 9). The balloon was then advanced from the groin toward the lesion, with contrast outlining its position (Figure 10). Following balloon angioplasty, the flush occlusion was successfully opened; however, a thrombus was noted at the origin of the profunda femoris artery (Figure 11).

**Figure 12:**
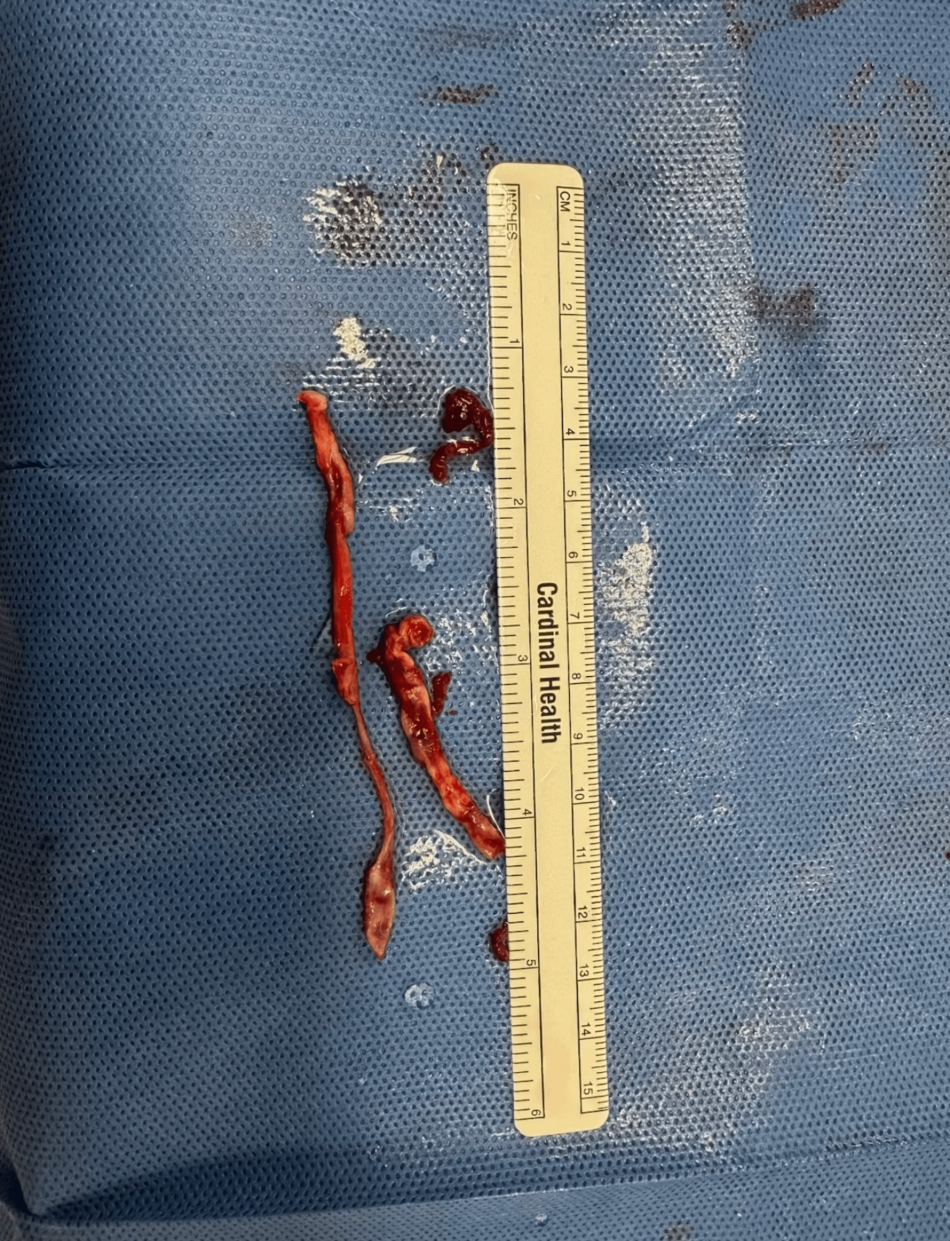
Extraction of thrombus from the left popliteal artery

Sequential balloon angioplasty was performed using a 6 mm × 200 mm plain balloon, followed by a 6 mm × 300 mm drug-coated balloon (Figure 8, Figure 9). Thrombus and atherosclerotic plaque were also extracted (Figure 12).

**Figure 13:**
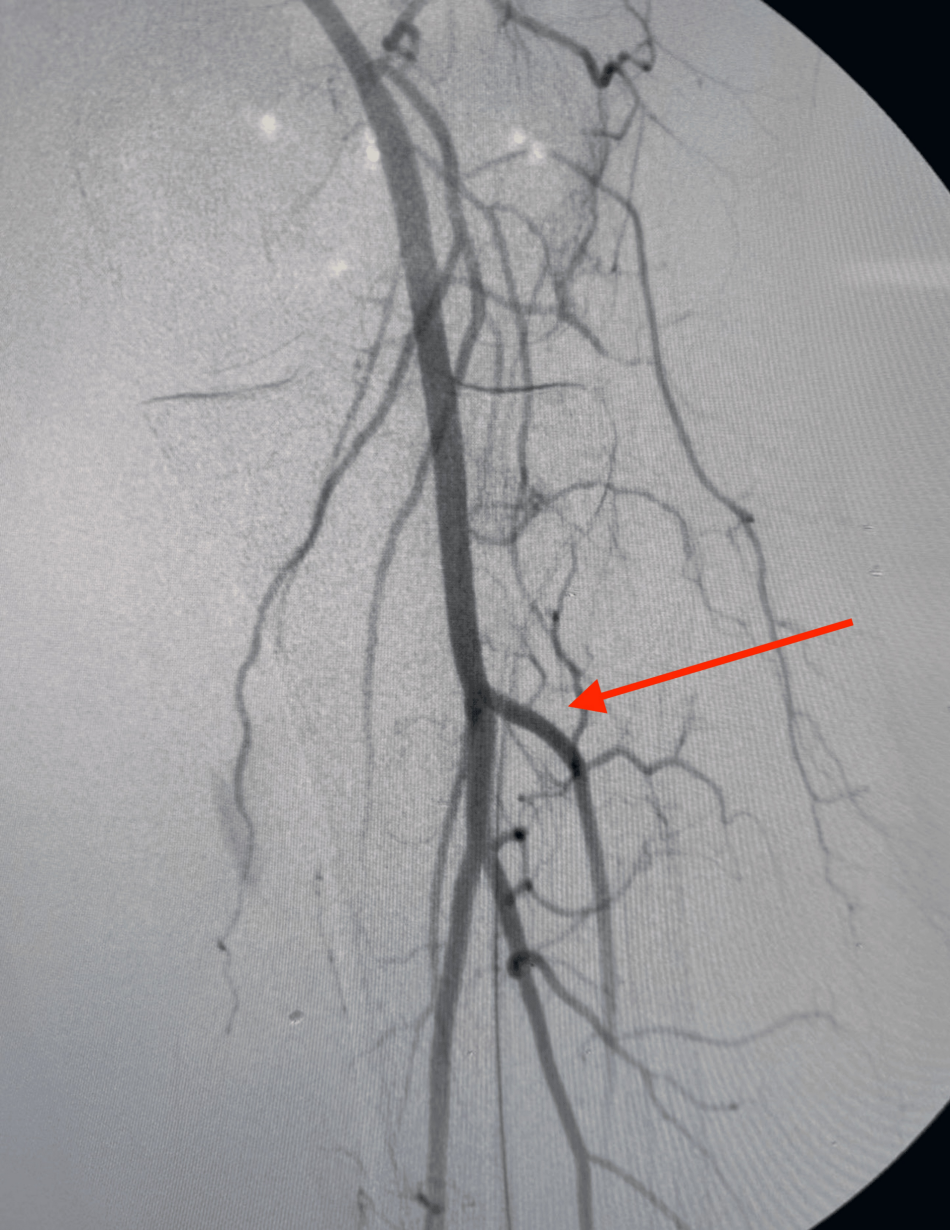
Fluoroscopic angiogram after retrograde popliteal angioplasty demonstrating restoration of flow through the left SFA (red arrow) SFA, superficial femoral artery

**Figure 14:**
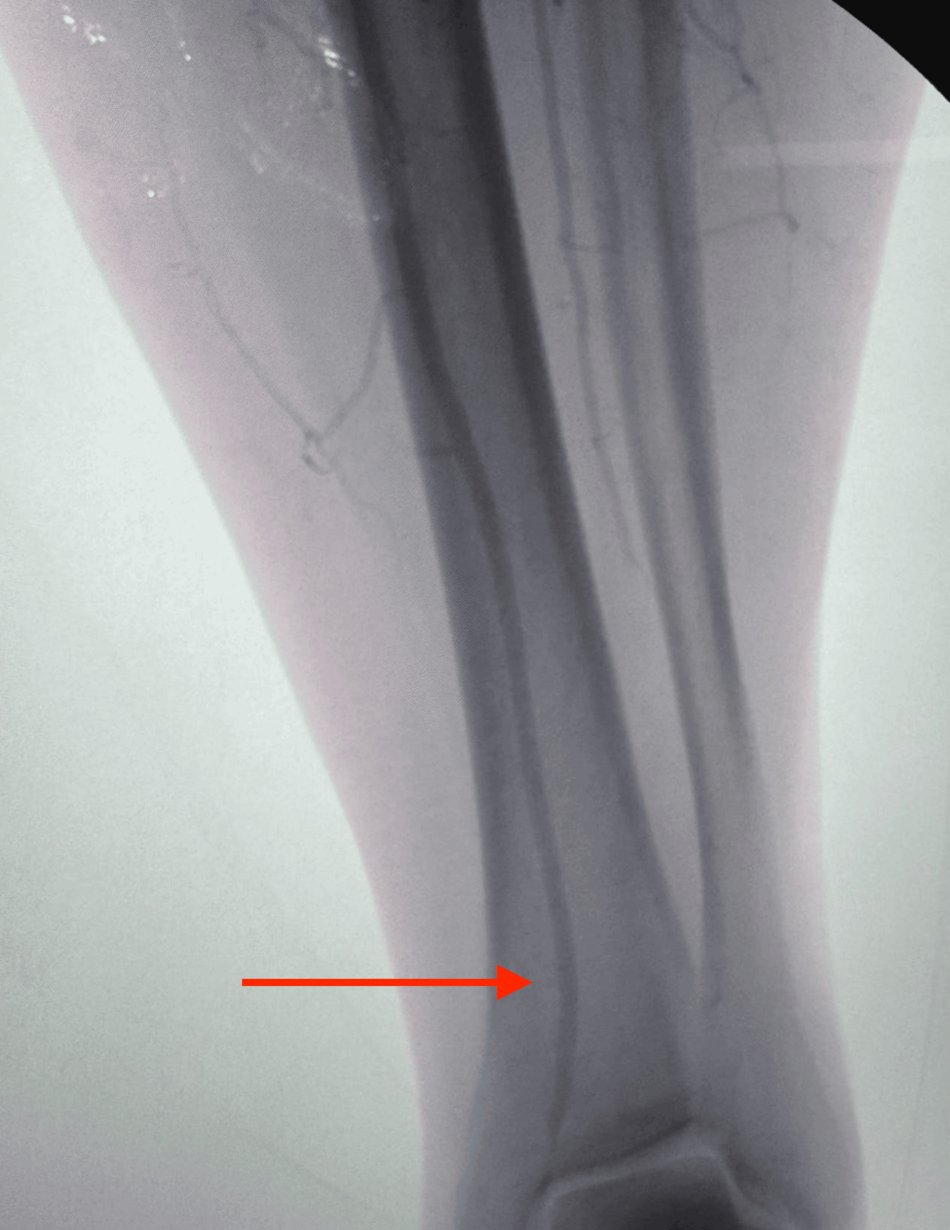
Guidewire successfully advanced distally to the level of the ankle (red arrow)

**Figure 15:**
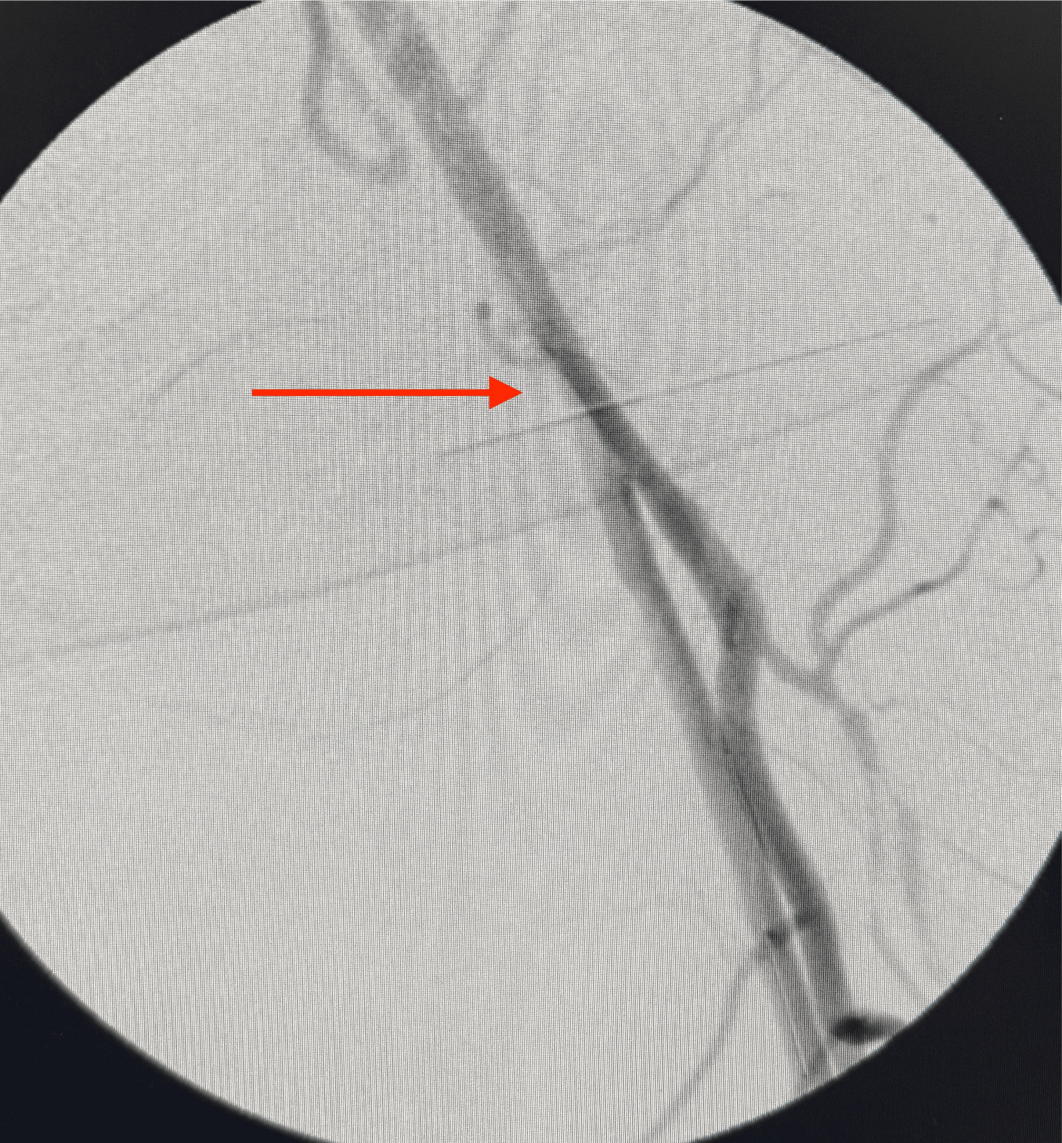
Selective catheter angiogram after retrograde popliteal angioplasty demonstrating restoration of flow (red arrow) through the left SFA and proximal profunda femoris artery SFA, superficial femoral artery

Retrograde popliteal angioplasty was completed, and fluoroscopic angiography demonstrated restoration of flow through the left SFA (Figure 13). The guidewire was successfully advanced distally to the level of the ankle (Figure 14). Post-procedure selective catheter angiography confirmed persistent patency of the left SFA and proximal profunda femoris artery (Figure 15).

**Figure 16:**
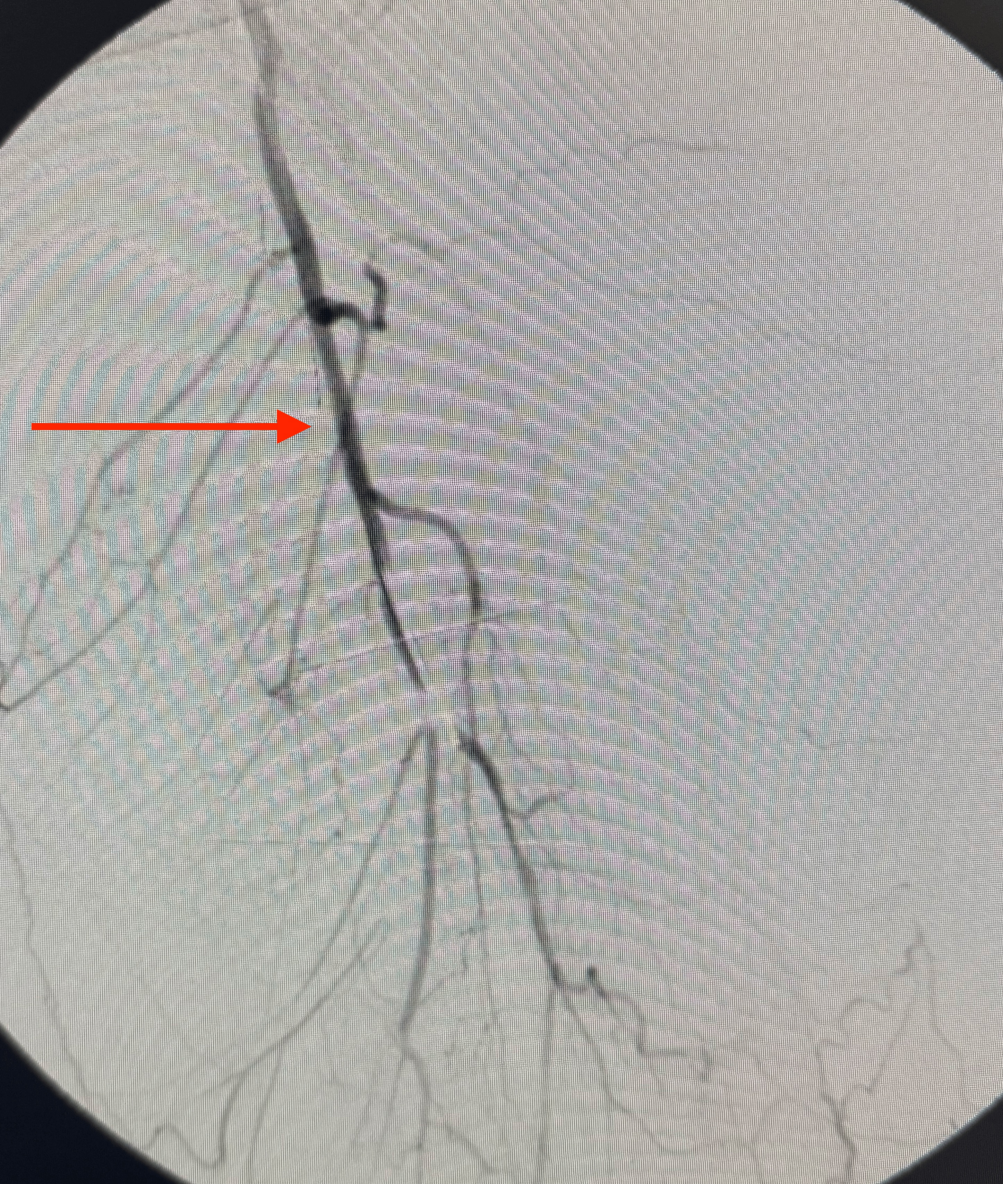
Follow-up selective angiogram demonstrating persistent patency of the lower portion of the profunda femoris artery (red arrow) after retrograde popliteal revascularization

On follow-up, selective angiography demonstrated persistent patency of the left SFA at the site of retrograde popliteal revascularization (Figure 16). Clinically, the patient had improved distal pulses in the affected extremity and complete resolution of ischemic pain.

All assessment tools, measurements, and procedural descriptions in this case report were developed by the authors.

## Discussion

Femoral-popliteal (fem-pop) bypass is an established revascularization strategy for SFA occlusions. While effective, it carries increased risks, including groin infection and lymphatic complications, particularly in patients with comorbidities [8]. In this patient, although fem-pop bypass could have been performed, the elevated risk of lymphatic complications made a less invasive approach preferable.

The retrograde popliteal approach was chosen for its direct access to the distal true lumen, enabling crossing of a chronic total occlusion of the SFA. This technique requires only a single incision, reducing surgical trauma and minimizing the risk of wound or lymphatic complications compared with fem-pop bypass [4,5]. Sequential balloon angioplasty using a Mustang and a Lutonix drug-coated balloon, combined with thrombus and plaque extraction, successfully restored flow, demonstrating the efficacy of this method in complex lesions that are difficult to cross antegradely [4,5].

Additionally, a hybrid endovascular strategy via the right common femoral artery was employed to remove thrombus from the profunda femoris artery. This allowed simultaneous treatment of both proximal and distal lesions while minimizing operative trauma, highlighting the versatility of combining retrograde popliteal access with complementary endovascular interventions [5,6].

In this patient, the combination of retrograde popliteal angioplasty and hybrid endovascular intervention resulted in complete revascularization, improved distal pulses, and resolution of ischemic symptoms. This case demonstrates that retrograde popliteal revascularization is a feasible and effective alternative to fem-pop bypass, particularly in patients with complex occlusions and comorbidities where minimally invasive strategies are preferable [4-6].

## Conclusions

Retrograde popliteal revascularization is a feasible and effective option for managing flush SFA occlusions, particularly when antegrade access fails. This approach can reduce the need for fem-pop bypass, thereby minimizing surgical risks such as groin infection and lymphatic complications. Furthermore, it allows for simultaneous or complementary endovascular interventions, such as thrombus removal from the profunda femoris artery, providing a hybrid strategy that maximizes revascularization success while minimizing operative trauma. This case demonstrates that retrograde popliteal access is a safe, less invasive alternative for complex peripheral arterial occlusions.
